# T Helper Cells Fate Mapping by Co-stimulatory Molecules and its Functions in Allograft Rejection and Tolerance

**Published:** 2014-08-01

**Authors:** R. Abdoli, N. Najafian

**Affiliations:** 1 Transplantation Research Center, Renal Division, Brigham and Women’s Hospital, Harvard Medical School, Boston, MA 02445, USA; 2 ***Cleveland Clinic Florida, 2950 Cleveland Clinic Blvd. Weston, FL 33331, USA***

**Keywords:** T cell receptor, Receptors, Antigen, B-Cell, Major histocompatibility complex, Transplantation, Antigens, differentiation, T-lymphocyte, Transplantation tolerance, T-Lymphocytes, helper-inducer, T-Lymphocytes, regulatory

## Abstract

T cell differentiation is dictated by a combination of T cell receptor (TCR) interaction with an antigen-bound major histocompatibility complex (MHC), and co-stimulatory molecules signal. The co-stimulatory signal can be positive or negative, and amplifying or diminishing the initial signal. However, the secondary co-stimulatory signal is not obligatory and its necessity is dictated, in part, by the stage of T cell development. In the field of transplantation, directing the T cell differentiation process can lead to therapeutic possibilities that promote allograft tolerance, and hinder unfavorable alloimmune responses. Therefore, understanding the details of T cell differentiation process, including the influence of co-stimulatory signals, is of paramount importance. It is important to note there is functional overlap between co-stimulatory molecules. It has been observed that some co-stimulatory signals have different effects on different T cell subsets. Hence, blockade of a co-stimulatory signal pathway, as part of a therapeutic regimen in transplantation, may have far reaching effects beyond the initial therapeutic intent and inhibit co-stimulatory signals necessary for desirable regulatory responses. In this review, co-stimulatory molecules involved in the differentiation of naïve T cells into T helper 1 (Th1), T helper 2 (Th2), T helper 17 (Th17), inducible regulatory T cells (iTregs), and T helper 9 (Th9) cells and their overlap are discussed.

## INTRODUCTION

Co-stimulatory pathways act synergistically to provide stimulatory and inhibitory signals that in combination with the T cell receptor-major histocompatibility complex (TCR-MHC) signal pathway activate naïve T cells. Activated naïve T cells are called effector cells. The effector cells may later develop into effector memory (EM) or central memory (CM) cells [1].

The co-stimulatory signals are critical for naïve T cell activation, and it has been shown that in the absence of these signals, TCR signal alone leads to T cell anergy, therefore, preventing an effective T cell response and promoting tolerance *in vitro* [2]. Knowledge of the co-stimulatory pathways is crucial in understanding the T cell immune response. The three major families of co-stimulatory molecules are immunoglobulin (Ig) superfamily, tumor necrosis factor-tumor necrosis factor receptor (TNF-TNFR) superfamily, and T cell immunoglobulin and mucin (TIM) superfamily [3-5] (Table 1). This review explores the role of co-stimulatory pathways in effector T helper cells functional differentiation during alloimmune response. 

**Table 1:** T cell lineages with their corresponding transcription factors, the cytokines they produce, their physiological functions and potential adverse effects, and the co-stimulatory molecules that can affect their activity by either promotion or inhibition of their lineage differentiation

**Lineage**	**Transcription Factors**	**Cytokine Production**	**Function/ Adverse Effect**	**Co-stimulatory Molecules**
Th1	STAT-1 STAT-4 T-bet	IFN-γ Lymphotoxin IL-2	Cellular immunity against intracellular pathogens Viral immunity Hypersensitivity/Autoimmunity	CD28; OX40; ICOS; CD40L; 4-1BB; TIM-1 TIM-3 (inhibitory); CTLA-4 (inhibitory); PD-1 (inhibitory)
Th2	STAT-5 STAT-6 GATA-3 IRF-4	IL-4 IL-5 IL-6 IL10 IL-13	Humoral immunity/B-cell help Extracellular parasitic/helminthic infections Allergy/Atopy	CD28; OX40; ICOS; TIM-1; TIM-4; TIM-2 CTLA-4 (inhibitory); PD-1(inhibitory)
Th17	ROR-T STAT-3 IRF-4 c-maf	IL-17A IL-17F IL-21 IL-22	Mediate responses to extracellular bacteria and fungi Autoimmunity	CD28; ICOS; TIM-1; CD40L; OX40 TIM-3 (inhibitory)
Th9	STAT-6 IRF-4	IL-9 IL-10	Helminthic infections Atopy/Autoimmunity	-
Treg	FoxP3	TGF-β IL-10 IL-35	Regulation/Suppression of the immune response/Tolerance	CD28; CTLA-4; PD-1/PDL-1; TIM-3; GITR; CD30 OX40 (inhibitory); TIM-1 (inhibitory)

**EFFECTOR T CELLS**

**Figure 1:**
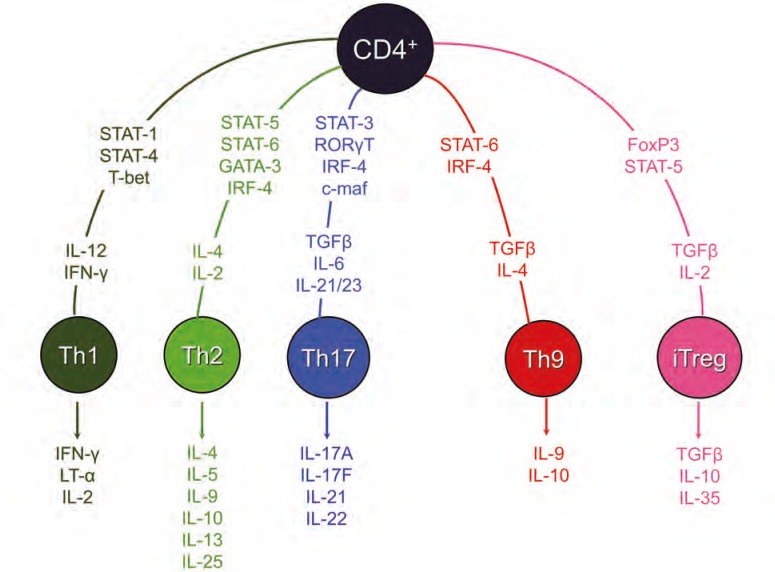
Schematic of helper T cell fate. The corresponding transcription factors and cytokines responsible for differentiation into T helper 1 (Th1), Th2, Th17, Th9, and iTregs are shown. The subsequent cytokines produced by differentiated helper T cells is also indicated

T helper cells, commonly identified by the expression of cluster of differentiation 4 (CD4) on their cell surface, are important contributors to the adaptive immune response. Therefore, they are key factors in autoimmunity, alloimmunity, and allergic reactions. To mount an appropriate immune response, T helper cells differentiate into various subsets. The differentiation process is dictated by a combination of the primary TCR-specific antigen-MHC signal and the secondary signals by co-stimulatory molecules. As a result, T helper cells can differentiate into various lineages including Th1, Th2, Th17, iTregs, and Th9 each producing specific sets of cytokines and having distinct functionality [3, 4, 6-9] (Fig 1, Table 2). In addition to the primary and secondary signals, the cytokines present also play a role in the differentiation decision [10]. Furthermore, it has been shown that the affinity with which the TCR binds to its specific antigen and the TCR signal strength are also important factors in determining the fate of the naïve T helper cells [10, 11]. It is also important to note that the differentiation process is not a terminal event and different CD4^+^ T cell subsets can mutually differentiate [12]. For example, under specific conditions Th17 and Tregs can interconvert [4, 13].

**Table 2:** Co-stimulatory molecules, their known ligands, their protein superfamily, and their expression patterns in leukocytes

**Co-stimulatory Molecule**	**Ligands**	**Family**	**Expression**
CD28	B7-1 (CD80); also binds PDL-1		
B7-2 (CD86)	IgG - CD28/B7	Constitutive - all naive CD4 & CD8 T cell subsets	
CTLA-4	B7-1 (CD80)		
B7-2 (CD86)	IgG - CD28/B7	Constitutive - Tregs	
Inducible - activated T cells			
ICOS	ICOS-L	IgG - CD28/B7	Inducible
PD-1	PDL-1; also binds B7-1		
PDL-2	IgG - CD28/B7	Constitutive - Tregs/Tfh	
Inducible - activated CD4/CD8, activated B cells, NK cells & macrophages			
CD27	CD70	TNF/TNFR	Constitutive - naive T, B & NK cells
Inducible			
CD30	CD30L	TNF/TNFR	Constitutive - Tregs
Inducible - activated T effector/memory			
CD40L	CD40	TNF/TNFR	Inducible - activated T cells, NK cells, eosinophils, platelets
OX40	OX40L	TNF/TNFR	Inducible - activated T cells
TIM-1	TIM-1		
TIM-4	TIM	Inducible - activated CD4 & CD8T cells	
TIM-2	Semaphorin 4A (Sem4A)	TIM	Inducible - activated T cells (Th2)
TIM-3	Galectin-9	TIM	Inducible - terminally differentiated Th1 cells
TIM-4	TIM-1		
	TIM	APCs	

**TH1 CELLS**

Th1 cells are the first group of differentiated CD4^+^ cells identified [14]. Th1 cells are mainly considered responsible for alloimmune response and allograft rejection in the context of transplantation [15, 16].

CD40 ligand (CD40L), also known as CD154, is a protein marker and a member of TNF-TNFR superfamily mainly found on the surface of activated Th1 cells [17]. CD40L is a co-stimulatory molecule that upon binding to CD40 on the surface of antigen presenting cells (APCs) induces the secretion of inflammatory cytokines TNF and IL-12 by T cells. This, in turn, leads to the activation of associated APCs by upregulating the expression of MHC, CD80, and CD86 in them [18]. CD40 expression is also upregulated in macrophages, dendritic cells, and B cells creating a positive feedback loop and further intensifying the antigen-specific signaling. Due to the central role of CD40L in Th1 lineage activation, interruption of the CD40-CD40L pathway leads to inhibition of Th1 inflammatory response [19]. Indeed, the use of anti-CD40L monoclonal antibodies (mAbs) or CD40L knock-out strains has been shown to drastically improve allograft survival, and prevent acute rejection in rodent and primate models [20-23]. Anti-CD40L mAbs have been used in combination with CTLA-4-Ig therapy and donor-specific transfusion, promoting tolerance [21, 24]. 

However, the beneficial effects of anti-CD40L seems to be counteracted when it is used in combination with immunosuppressive agents cyclosporine A (CsA), and methylprednisolone [25]. This is thought to be due to the down regulation of CD40L by the above-mentioned immunosuppressive agents [25]. On the other hand, it has been shown that rapamycin (sirolimus) has significant synergy with anti-CD40L, together leading to indefinite graft survival by reducing the frequency of alloreactive IFN-γ secretion and inhibition of chronic rejection [26]. 

The use of anti-CD40L antibodies in non-human primates and phase I clinical trials has been unsuccessful leading to high incidence of thrombotic complications due to the interruption of the CD40L signaling in platelets [27, 28]. Therefore, more recent efforts have focused on developing T cell-specific monoclonal anti-CD40L antibodies with promising early transplantation results [29]. 

In addition to CD40L, TIM-1 is another cell surface marker that is rapidly upregulated upon activation of naïve CD4^+^ T cells [30, 31]. Binding of TIM-1 to its ligand, TIM-4, on APCs sends a positive co-stimulatory signal to T cells [32]. TIM-1 is shown to control the differentiation of several Th subsets [33, 34]. However, following differentiation, TIM-1 expression is only maintained in Th2 cells while Th1 and Th17 cells cease to express this cell surface marker [31, 35]. In a multiple sclerosis mouse model, the use of a high affinity agonistic anti-TIM1 mAb led to enhanced Th1 and Th17 responses, suggesting that TIM-1 is involved in Th cell differentiation [33]. Moreover, the study of an antagonistic anti-TIM-1 antibody in fully MHC-mismatched murine cardiac transplant models led to improved survival, suggesting the inhibition of alloreactive Th1 cells and further confirming the role of TIM-1 in T helper cell differentiation [36]. 

In another study, recombinant human TIM-1 extracellular domain fusion protein (TIM-1-Fc) was successfully used as an immunosuppressive agent to significantly prolong fully MHC-mismatched murine cardiac allograft survival [32]. In this case, TIM-1-Fc inhibited the differentiation of CD4^+^ T cells into Th1 cells by reducing the AKT and ERK1/2 phosphorylation in the downstream intracellular signal transduction pathway necessary for Th1 activation [32]. Interestingly, by downregulating the Th1 response, TIM-1-Fc indirectly shifts the balance towards Th2 and nTregs, further prolonging allograft survival [32]. 

CD152, also known as CTLA-4, is another cell surface receptor that is upregulated in activated T cells [37]. CTLA-4 is structurally related to CD28, a well-studied molecule that provides co-stimulatory signals in T cells, and binds to the same ligands, B7-1 and B7-2, on antigen presenting cells [2].

CTLA-4 binds to CD28 receptors with much greater affinity and inhibits the downstream Akt pathway, preventing the co-stimulatory signal necessary for IL-2 production, and T cell growth and proliferation [38, 39]. Therefore, CTLA-4 has been used in research to suppress Th1 and Th2 and inhibit their differentiation [40]. Qurenshi demonstrated that CTLA-4 expressing cells transendocytose and degrade B7-1 and B7-2 from other cells [41]. This can be another mechanism by which CTLA-4 prevents CD28 ligation and co-stimulatory signal, making it a good candidate for immune regulation. 

CTLA-4-Ig, a fusion protein consisting of the extracellular CTLA-4 domain and Fc constant portion of IgG1, has been used in combination with tolerance induction by donor splenocyte transfusion to effectively prolong allograft survival in murine models [42-44]. Furthermore, CTLA-4-Ig can be used in combination with other immunosuppressants such as rapamycin, calcineurin inhibitor, and methylprednisolone in rodent transplant models [26]. It is interesting to note that much of what is known about the role of CD28 signaling in transplantation has been obtained through studies using CTLA-4-Ig. In human transplant recipients, belatacept, a higher affinity derivative of CTLA-4-Ig has been approved. However, some of the initial transplant trials have demonstrated complications in the form of higher rates of severe acute rejection [45]. 

TIM-3 is a cell surface marker present on cells of innate and adaptive immune system [35, 46, 47]. Galectin-9 is TIM-3 receptor found in various cell types including regulatory T cells and naïve T helper cells [49]. It has been shown that once T helper cells get activated and differentiate into Th1 lineage, they express TIM-3 [47, 48]. IFN-g leads to the upregulation of Galectin-9, which binds to TIM-3 to create a negative co-stimulatory signal promoting Th1 cell death through apoptosis and necrosis [49]. As expected, it has been shown that TIM-3 blockade accelerates Th1-mediated autoimmune disease in rodent models [48]. Similarly, TIM-3 blocks the secretion of Th1 and Th17 cytokines in human studies, although in this case TIM-3 does not appear to induce cell death [50]. We have shown that blocking TIM-3 by using RMT3-23 blocking antibody accelerates allograft rejection in the absence of CD80/86 and CD28 co-stimulatory signaling [51]. This is expected as in the absence of TIM-3 inhibitory signal, allospecific CD4^+^ T cells expand and polarize to Th1 cells leading to increased secretion of IL-6, IL-17, IFN-g, and Granzyme B cytokines [51].

Programmed death 1 (PD-1) protein with its ligand (PDL-1) are involved in inhibitory and regulatory pathways that promote peripheral tolerance and engraftment [52-54]. It has been shown that PDL-1 blockade leads to increased rate of differentiation of alloreactive Th1 cells, hence, its therapeutic potential in transplantation [53, 55]. Furthermore, B7-1 surface protein is also able to bind to PDL-1 to initiate an inhibitory signal pathway [56].

Finally, in addition to CD28 and TIM-1, OX40 is also a cell surface protein that is known to create a positive signal to promote Th1 differentiation and IL-12 and IFN-a proinflammatory cytokines production [10, 33, 57].

**TH2 CELLS**

There are opposing views on the role of Th2 cells in the alloimmune response. While some data indicate that Th2 cells are involved in the rejection response [58, 59], others suggest that they may have a regulatory role [60].

The strength of the TCR signal is essential in determining the fate of undifferentiated helper T cells. A weak TCR signal in combination with CD28 co-stimulatory signal promotes IL-4 production and activation of the GATA-3 pathway leading to differentiation into Th2 cells [10, 61, 62]. On the contrary, a strong TCR signal promotes IFN-g production and Th1 cell differentiation [10]. As discussed before, in addition to the main TCR-MHC signal, co-stimulatory signals also play an important role in determining T cell fate and differentiation. The use of agonistic anti-CD28 antibodies in rodent models has led to the prolongation of allograft survival [63]. In addition, a human-primate chimeric mAb that selectively blocks CD28 co-stimulatory signal has been shown to inhibit both acute and chronic rejection in non-human primates [64], emphasizing the importance of the co-stimulatory signals in the T-cell mediated immune response.

OX40 is another co-stimulatory molecule that provides the signal for the differentiation of naïve T helper cells to Th2 cells. The signal initiates the downstream cellular pathway that leads to the activation of nuclear factor of activated T-cells, cytoplasmic 1 (NFATC1) [65]. OX40 acts in concert with CD28 to prompt the production of IL-4, a cytokine that induces the differentiation of naïve CD4^+^ cells to Th2 cells [65]. Indeed, in the absence of CD28, the Th2 response is weakened and the naïve Th0 cells differentiate into Th1 cells due to the presence of IFN-a and IL-12 [57, 61]. 

OX40 has also been shown to promote *in vitro* Th2 differentiation in human naïve T cells, although it did not inhibit upregulation of IL-12 and IFN-g [66]. Similarly, the use of anti-OX40 mAb in a murine cardiac transplant model accelerated rejection by both Th1 and Th2 responses [67]. The blockade of OX40-OX40L signaling with anti-OX40L mAb in combination with rapamycin resulted in significant improvement in rodent allograft survival compared to rapamycin alone confirming the role of this molecule in Th2 response [68]. In addition, the use of anti-OX40L mAb in combination with CD28-B7 blocking antibodies prolongs both cardiac and skin graft survival in murine models [68, 69].

Inducible T cell co-stimulator (ICOS) is a cell surface protein that is upregulated in activated T cells upon CD28 co-stimulation, and conversely downregulated following CTLA-4 signaling [70]. Binding of ICOS to its ligand, ICOS-L, creates a feedback loop that downregulates CD86, the ligand of CD28 and CTLA-4 on APCs [70]. It has been shown that ICOS is able to provide the necessary co-stimulatory signals for T cell activation in the absence of CD28 and hence, regulates differentiation into Th1, Th2, and Th17 cells [71, 72, 75, 76]. However, ICOS signaling seems to be most crucial in Th2 cell differentiation by enhancing the IL-4R signal [73]. ICOS-deficient patients are also deficient in Th1, Th2, Th17, and central memory cells further confirming the role of this cell surface immunoglobulin in T cell polarization [74]. Similarly, ICOS expression is upregulated in acute and chronic allograft rejections [72]. Therefore, ICOS blockade has been used as a therapeutic method to hinder the immune response and prolong fully MHC-mismatched murine allograft models [71, 72]. The use of ICOS blockade at a later time point leads to best results in terms of prolonging graft survival [71], which is consistent with earlier findings that ICOS is only expressed after T cell activation. Additionally, STAT-4 knockout mice that miss this crucial transcription factor for Th1 cell activation still show prolongation of graft survival upon administration of ICOS blockade, suggesting ICOS effect on Th2 cell activation [71]. ICOS blockade can be used in concert with CD40L to prevent chronic rejection [72]. Also, the use of ICOS blockade with a short course of CsA leads to permanent engraftment of fully MHC mismatched cardiac allografts with normal histology at day 100 [72].

TIM-1 and TIM-4 signaling is also involved in Th1, Th2, and Th17 differentiation pathways and proliferation. The specific affinity antibody to certain TIM-1 epitopes used as well as signal strength dictates TIM-1 role in the differentiation decision [33]. For example, in experimental autoimmune encephalomyelitis models, the use of a high affinity agonistic antibody intensified the severity of the disease by promoting Th1 and Th17 responses, while a low affinity antibody repressed disease development by promoting Th2 response [33]. Furthermore, *in vivo* allergic responses following exposure to *Staphylococcus enterotoxin* B or cholera toxin and peanut extract lead to the upregulation of TIM-4 surface marker on intestinal and bone marrow dendritic cells respectively that subsequently lead to Th2 differentiation and allergic response [77, 78]. However, more data are needed to clarify TIM-4 role in transplantation.

Finally, activated murine CD4^+^ T cells that are mainly of the Th2 phenotype express a cell surface marker known as TIM-2 [79]. To date, TIM-2 has only been found in mice and it is hypothesized to be involved in negatively regulating Th2 cells during allergic and autoimmune responses, with little known about their role in transplantation [79].

**TH17 CELLS**

Th17 cells can trigger the rejection response in transplantation independent of Th1 proinflammatory lymphocytes and IL-17A cytokine [80, 81]. However, many studies have shown that proinflammatory cytokine, IL-17A, secreted by cells other than Th17 is a major contributor to the rejection response [82]. Our group was able to demonstrate that Th17 cells can mediate acute cardiac rejection and vasculopathy in murine transplant models independent of Th1 [80]. Nevertheless, the role of Th17 cells in normal alloimmune response directed mainly by Th1 cells is to be determined.

CD28 co-stimulatory signal is necessary for Th17 differentiation, as with any other helper T cell subset [17, 76]. This has been confirmed by the study showing that the use of anti-CTLA-4 and CTLA-4-Ig that respectively, potentiate and block CD28 co-stimulatory signal, leads to the augmentation or inhibition of Th17 differentiation [83]. However, another study demonstrated that CTLA-4-Ig blockade of CD28 co-stimulation facilitated Th17 differentiation in both mouse and human models, and CD28 signal activation by its binding to anti-CD28 mAbs inhibits the differentiation of naïve CD4^+^ cells into Th17 [51, 84].

Similar to CD28, ICOS is involved in Th17 cell differentiation as shown both *in vivo* and *in vitro* [76]. ICOS co-stimulatory signal has been shown to prompt IL-17 secretion by Th17 cells [76]. Additionally, ICOS signaling induces the transcription factor c-Maf which enhances IL-21 secretion leading to expansion and maintenance of Th17 cells [85].

In murine models, ICOS is not a prerequisite for Th17 differentiation; however, it is necessary for maintenance of differentiated Th17 cells [85]. On the other hand, ICOS co-stimulatory signal is necessary for both differentiation and maintenance of human Th17 cells [86]. Indeed, ICOS-deficient patients show decreased number of CD4^+^ effector Th1, Th2, and Th17 cells, as well as central memory cells [74].

In addition to triggering Th1 and Th2 differentiation, TIM-1 signaling also plays a role in Th17 differentiation. This was shown by the use of agonistic anti-TIM-1 mAb that trigger the downstream TIM-1 signaling pathway leading to the conversion of regulatory T cells to Th17 cells, and therefore, inhibiting tolerance development in islet transplantation models [34]. Additionally, the use of anti-TIM-1 to block TIM-1 signaling leads to tolerance by inhibition of IL-17 producing cells in a CD28 and CD40L-independent manner [87]. 

TIM-4, another member of the transmembrane immunoglobulin and mucin domain family, prevents differentiation of naïve T cells to Th17 and IL-17 production by differentiated Th17 cells [88]. TIM-3 signaling also regulates the immune response leading to allograft tolerance [51]. Blocking TIM-3 by using RMT3-23 blocking antibody accelerates allograft rejection in mouse cardiac transplant models independent of CD28 and CD80/CD86 co-stimulatory signals [51]. This is contributed to increased rate of differentiation of effector Th1, Th17, as well as IL-6, IL-17, IFN-g, and Granzyme-β-producing cells [51]. Similarly, TIM-3 inhibits secretion of proinflammatory cytokines by Th1 and Th17 cells in human studies [50].

In addition to the previously discussed role in Th1 differentiation, CD40-CD40 ligand pathway is also involved in Th17 cell differentiation [17]. The CD40-CD40L pathway creates the perfect setting for Th17 cell differentiation by stimulating IL-6 and transforming growth factor beta (TGF-b) secretion by dendritic cells and macrophages, respectively [89]. In dendritic cells, stimulation of pattern recognition receptors by autoantigens leads to stimulation of these cells to produce IL-6 during an autoimmune response [89]. It has been shown that CD40 knockout mice are unable to build a normal Th17 response against self-antigens and are immune from experimental autoimmune encephalomyelitis (EAE) [89]. 

Finally, the effect of OX40 receptor on Th17 cells is unclear. On the one hand, *in vitro* studies suggest that OX40 signaling inhibits Th17 cell differentiation through increased secretion of IFN-g and IL-4 [90]. However, *in vivo* studies show that OX40 signaling is required for Th17 activity as seen by facilitation of EAE and rheumatoid arthritis [91, 92].

**TREGS**

Natural regulatory T cells (nTregs) are a subpopulation of T cells that are formed in the thymus. Alternatively, induced Tregs (iTregs) may be generated in the periphery from CD4^+^ cells in the presence of TGF-b [7]. Tregs are most commonly characterized by the expression of forkhead box P3 (FoxP3) transcription factor. They are primarily responsible for controlling the immune response. Tregs are critical to the development and maintenance of self-tolerance, and they can eliminate autoimmune disease. In the context of transplantation, Tregs are vital to the induction and maintenance of allograft tolerance.

Mice deficient in CD28 or its ligands have a significantly reduced numbers of natural Tregs [93-96]. Therefore, CD28 co-stimulatory signals appear to be critical to the development of nTregs in the thymus [93]. CD28 co-stimulation also leads to the production of IL-2, which can further direct the differentiation of CD4^+^ T cells into CD4^+^ CD25^+^ Tregs [97, 98]. On the other hand, high levels of CD28 co-stimulation have the opposite effect by inhibiting the generation of iTregs and promoting the differentiation of effector T cells through intracellular signaling pathways involving lymphocyte-specific protein tyrosine kinase (LcK) [99]. 

It has been shown that CD28-deficient mice undergo accelerated rejection of single MHC class II-mismatched cardiac allografts [96].This observation has been attributed to an increase in the number of Th1 and Th2 effector T cells and their associated cytokines, and a decrease in the number of Tregs [96], signifying the immunoregulatory role of CD28. However, CD28^–/–^ mice show a prolongation of cardiac allograft survival in fully MHC-mismatched transplant models indicating plasticity in CD28 role in the immune response [96, 100]. Although the use of agonistic anti-CD28 mAb leads to activation and expansion of functional Tregs both *in vitro* and *in vivo* in rodent models, the same therapy had completely opposite results in human trials resulting in massive cytokine production in phase I clinical trials of six healthy volunteers after administration of agonistic anti-CD28 mAb [101-103]. 

As discussed previously, CTLA-4 (CD152) is inducibly expressed on T cells and competes with CD28 for interaction with B7.1 and B7.2 on APCs, providing a negative co-stimulatory signal in Th1/Th2 cell differentiation pathways. Additionally, CTLA-4 is constitutively expressed on Tregs and has a central role in regulatory T cells stability and function [2]. The effect of the absence of CTLA-4 was studied in CTLA-4 deficient mice [2]. In these mice, environmental antigens activate CD4^+^ cells irrepressibly, leading to overwhelming lymphocyte proliferation and eventually inflammation [2]. 

CTLA-4 controls the inflammatory response by both intrinsic and extrinsic cell mechanisms. The cell intrinsic mechanism involves the inhibition of Erk and Akt phosphorylation pathways [104]. However, there are also data showing that CTLA-4 exerts its immune inhibitory and tolerance inducing effects by activating an enzyme called indoleamine 2,3-dioxygenase (IDO) after binding to its B7 ligand [104]. The cell extrinsic mechanism of regulating immune response in CTLA-4 expressing cells involves the binding and trans-endocytosis of B7.1 and B7.2 on APCs; therefore, making them unavailable for CD28 interaction and eventual immune response activation [41]. 

Belatacept is a mutant, higher affinity form of the CTLA-4 immunoglobulin that has been approved for use in transplantation [105]. Belatacept binds to B7.1 and B7.2, blocking their interaction with both CD28 and CTLA-4. As a result, belatacept not only inhibits CD28 signal transduction and activation pathways, but also inhibits the CTLA-4 interaction with B7 preventing inhibitory cell intrinsic signaling. This might explain the observation of an increase in early acute rejection in phase III belatacept trials in the group that received the treatment [45]. Our group demonstrated this dual effect by showing that hCTLA4-Ig inhibits natural Tregs generation leading to the acceleration of cellular rejection in MHC class II mismatched models in naïve mice; however, it also prevents rejection in fully MHC-mismatched cardiac transplant mice [106]. 

Additionally, Bluestone has shown that a cohort of renal transplant patients treated with anti-IL-2R and belatacept or calcineurin-inhibitor therapy experienced a short-term reduction in the number of circulating Tregs that can be attributed to anti-IL-2R treatment [107]. It is also worth mentioning that, in addition to CTLA-4, the PD-1–PDL-1 pathway also exerts immune inhibitory effects by blocking the Akt phosphorylation and signal transduction pathways, and enhancing the phosphatase and tensin homolog (PTEN) cell cycle regulator leading to differentiation and maintenance of CD4^+^ CD25^+^ FoxP3^+^ Tregs [108, 109]. 

TIM-3–Galectin-9 interaction also controls regulatory T cell activation. Natural Tregs constitutively express TIM-3 as a cell surface marker in mouse cardiac transplant models [51]. Galectin-9 is universally expressed on both naïve helper T cells and non-activated regulatory T cells; however, once activated, helper T cells down-regulate their Galectin-9 expression while no significant difference in expression is observed in regulatory T cells [47, 48]. 

To confirm the role of TIM-3 in regulatory T cell activation, TIM-3 has been blocked to show a significant decrease in the number of allospecific Tregs, as well as a decrease in their suppressive capacity, resulting in significant decrease in peripheral immune-tolerance [47, 48]. Similarly, it has been shown that adding soluble Galectin-9 leads to survival prolongation of both skin and cardiac allograft models, further confirming the role of TIM-3–Galectin-9 signal in regulatory T cells activation [110, 111]. The prolongation of allograft survival is due to a decrease in Th1 and Th17 cytokine secretion and proliferation, as well as an increase in the number of regulatory T cells [111]. 

TIM-1 receptor, on the other hand, is responsible for the differentiation of effector Th1, Th2, and Th17 cells, while suppressing the differentiation and immunosuppressive function of Tregs [31, 33, 34]. Indeed, use of anti-TIM-1 antibody has been shown to prevent transplant tolerance in murine islet transplantation models by enhancement and suppression of the effector and regulatory T cell functions, respectively [34].

Finally, CD134, also known as OX40, is a receptor that inhibits TGF-b, a cytokine responsible for the differentiation of CD4^+^ naïve T cells and effector T cells into CD25^+^ FoxP3^+^ Tregs [112, 113]. In addition, OX40 signaling in Tregs has been shown to decrease their immunosuppressive and immunoregulatory function [113, 114]. However, studies of OX40 knockout mice found no difference in Tregs number or suppressive function compared to the wild type [113]. 

**TH9 CELLS**

Th9 is a more recently discovered subset of T cells. Cytokines, TGF-b, and IL-4 are involved in directing the differentiation into Th9 cell line [6, 9]. It is suggested that TGF-b reprograms Th2 cells to become Th9 [9]. Another study shows that, in the presence of IL-4, TGF-b induces Th9 rather than Treg cell differentiation [6]. Furthermore, the transcription factors IRF-4 and STAT-6 appear to be required for Th9 cell differentiation [6, 115]. Th9 cells produce IL-9 and IL-10 cytokines and promote inflammatory response in tissues [6, 9]. Th9 cells are also involved in inflammatory responses occurred in asthma and autoimmune diseases [115]. However, the exact mechanism for Th9 cells differentiation and their role in transplantation is still unknown and awaits further investigation.

## CONCLUSIONS

Co-stimulatory molecules are critical components of the immune response. Co-stimulatory signals often work in concert and many co-stimulatory pathways are involved in multiple cellular signaling, exponentially adding to the complexity of T cells fate. Co-stimulatory molecules have great potential as therapeutic targets in transplantation, given their ability to alter and dictate the immune response. However, because of the complex nature of the co-stimulatory signaling, blockade of a co-stimulatory molecule may have consequences beyond the expected therapeutic intervention that needs to be taken into consideration in development of novel treatments.
